# Improvement of magnetite adsorption performance for Pb (II) by introducing defects

**DOI:** 10.3389/fchem.2023.1137246

**Published:** 2023-02-24

**Authors:** Yuxin Li, Guocheng Lv, Hao Liu, Xin Liu, Libing Liao

**Affiliations:** ^1^ Engineering Research Center of Ministry of Education for Geological Carbon Storage and Low Carbon Utilization of Resources, Beijing Key Laboratory of Materials Utilization of Nonmetallic Minerals and Solid Wastes, National Laboratory of Mineral Materials, School of Material Sciences and Technology, China University of Geosciences (Beijing), Beijing, China; ^2^ School of Science, China University of Geosciences (Beijing), Beijing, China

**Keywords:** magnetite, adsorption, oxygen vacancy, cation vacancy, Pb (II)

## Abstract

Surface defect engineering is an efficient strategy to enhance the adsorption properties of materials. After calcination in argon, the adsorption capacity of natural magnetite to Pb (II) is significantly improved. The Rietveld refinement, Mössbauer spectrum, and XPS were used to prove the existence of oxygen and cation vacancies in the crystal structure of magnetite after calcination, and it is found that the vacancy content is linearly related to the adsorption amount of Pb (II). This indicates that the increase in the adsorption performance of magnetite after calcination is determined by the vacancy. The adsorption capacity increases from 8 to 26 mg/g when the calcination temperature reaches 700°C. The equilibrium adsorption process of Pb (II) on magnetite can be well fitted to the Langmuir model, and the kinetic adsorption followed a pseudo-second-order mechanism. The improvement of the adsorption performance of magnetite is mainly due to the change in its structure, which depends on the oxidation degree and surface effect of magnetite in the calcination process. This work also provides a theoretical basis for the broad application of magnetite as environmental material.

## 1 Introduction

Magnetite crystallizes in the so-called inverse spinel structure (Space group, Fd3m). The chemical formula is often written as [Fe^3+^]_A_ [Fe^3+^, Fe^2+^]_B_ O_4_. This formulation shows that O atoms form a closed-packed cubic lattice, Fe^3+^ ions are located in the tetrahedral sites, and a 1:1 mixture of Fe^2+^ and Fe^3+^ ions fill the octahedral sites. The rapid electron hopping process occurs between Fe^2+^ and Fe^3+^ ions (Noh et al., 2015; Schöttner et al., 2019). Magnetite is a metal-deficient oxide with high electronic conductivity under high oxygen pressure (Castle and Surman, 1969; Nakamura A, et al., 1978). The self-diffusion coefficient of oxyanion is far less than that of cations. The non-stoichiometric feature of magnetite is the existence of cation vacancies in its structure. This is caused by the oxidation of Fe (II) at octahedral sites to Fe (III), which leads to an imbalance of charge, thus removing additional Fe (II) from the structure (Cervellino et al., 2014). The concentration of point defects in metal oxides is usually controlled by introducing a small number of foreign ions with different valences or changing the partial pressure of oxygen. (Nakamura et al., 1978). In binary oxide crystals, by introducing an appropriate external gas atmosphere, the defect concentration can be changed in a certain way. According to the reaction between oxygen and magnetite, cation vacancy is formed (Becker and Litterst, 1990). With the increase in temperature, the distribution of cation vacancies will also change, which researchers have learned using the Mössbauer spectroscopy (Wiβmann et al., 1998). In the temperature range of 1,100°C–1,400°C, the shape of the spectral line is affected by the cation diffusion process. Also, the exact lattice parameters and stoichiometry of nano ferric oxide can be used to determine the vacancy distribution of magnetite by synchronous X-ray scattering and Debye function analysis (Cervellino et al., 2014). The results show a small number of vacancies at tetrahedral stations, which is at most 3%–4% of the total. When magnetite nanoparticles are oxidized, the cation diffusion comes from removing iron ions from the octahedral site.

The defects in magnetite are not only cationic vacancies but also oxygen vacancies (Castle and Surman, 1969; Millot and Niu, 1997). It is well known that oxygen vacancies usually exist in metal oxides, especially in the oxidation process of some variable valence metal oxides (Lv et al., 2011; Greiner et al., 2012; Choudhury et al., 2015). Oxygen will also diffuse in Fe_3_O_4_ and during the diffusion process, oxygen defects will be generated under low oxygen pressure, and cation and anion vacancy pairs will be developed under the condition of high oxygen pressure, namely, Schottky defects (Vaari, 2015). Under reducing conditions, the formation of oxygen vacancies on the surface leads to reduced iron nearby (Santos-Carballal et al., 2014). In fact, oxygen vacancies created by ultra-high vacuum annealing are likely to reduce ferric iron to divalent iron (Rioult et al., 2016).

Magnetite is a common component in soil and sediment and has high adsorption capacity for dissolved metal ions such as Pb (II) (Bargar et al., 2004). It is increasingly used in groundwater pollution and soil remediation (Filip et al., 2014). Its surface defects are widely regarded as the active site of the reaction process (Gorski et al., 2010; Li et al., 2014; Lesiak et al., 2019). Much adsorptive behavior between contaminant and adsorbent is strongly affected by surface defects (such as point defects and step edges) of the adsorbent (Li et al., 2015; Yong et al., 2021; Li et al., 2022). Although calcination will cause aggregation of some materials, this phenomenon usually occurs on nanoparticles (there are interactions between nanoparticles) (Ding et al., 2009), it can still be used to introduce defects in micron-scale materials to improve the reaction activity in this paper and avoid aggregation reaction. Bui et al. engineered Fe_3_O_4_ surface defects by doping 1% mol Cr without affecting the magnetic properties of Fe_3_O_4_, and experimental analysis showed that defect control significantly impacted the photocatalytic performance of Fe_3_O_4_, making them efficient photocatalysts (Bui et al., 2020). Kim S et al. calcined magnetite in a reducing atmosphere for the decomposition of CO_2_ to effectively reduce CO_2_ in the atmosphere. With the increase of reduction temperature, the number of defect sites on the sample surface increased, providing reaction sites for decomposing CO_2_ (Kim et al., 2012).

There are abundant iron oxide minerals in nature, which have high adsorption and removal capacity of Pb (II) and affect the migration of Pb (II) on the Earth (Liang et al., 2017). Calcination is an effective strategy for introducing defects in the crystal structure (Nandi and Das, 2019; Li et al., 2020b), so we calcined magnetite in an inert atmosphere to improve the removal effect of magnetite on Pb (II) from water. In this work, we have done deep work with calcined magnetite in argon to study the changes in oxygen vacancy and iron vacancy. Natural magnetite was used as the precursor to obtain a mineral material with more defects to remove Pb (II) from water. The adsorption process was studied by equilibrium and kinetic adsorption experiments, and the effect of vacancies on adsorption capacity was invested.

## 2 Experimental details

### 2.1 Materials and reagents

The lead nitrate used in the experiment was purchased from Beijing Chemical Plant. A stock solution of Pb (II) was prepared by dissolving 1.599 g of solid Pb (NO_3_)_2_ (AR grade) in 1 L of deionized (DI) water. The natural magnetite was selected from Jinling Iron Mine in Zibo, Shandong Province. The sample was sieved through 200 mesh and then was prepared by magnetic separation to remove the impurities. And the obtained raw magnetite was named after Mag-25. After that, the obtained high-purity magnetite powder sample was calcined at 300°C, 500°C, 700°C, and 900°C in a tube furnace in argon at a heating rate of 5°C/min for 2 h and the samples were denoted as Mag-300, Mag-500, Mag-700, and Mag-900, respectively. All the chemicals used were analytical reagent grade without further purification, and DI water was used in all experiments.

### 2.2 Characterizations

The phase composition and crystal structure of the samples were affirmed by X-ray diffraction (XRD) on an X-ray powder diffractometer (D8 Advance, Bruker, Germany) with Cu *Kα*1 (*λ* = 1.5406 Å) radiation at 40 kV and 100 mA, a scanning speed of 1.2° 2*θ*/min, and a step size of 0.02° 2*θ* from 5° to 130°. Scanning electron microscopy (SEM, ZEISS, sigma 300, Germany) was used to characterize the morphologies of the samples. X-Ray fluorescence spectrometer (XRF, Thermo electron corporation, ARLADVANT X, United States) was used to determine the chemical composition of the sample. The Brunauer-Emmet-Teller (BET) specific surface area was determined from N_2_ adsorption-desorption isotherms using an automated gas sorption instrument (Micro Active for ASAP 2460, United States). The removal amount of Pb (II) was measured and calculated using an Inductively coupled plasma spectrometer (ICP-OES, Thermo scientific, iCAP 7,600, United States). The electron paramagnetic resonance (EPR) measurements were performed on a Bruker EMX plus model spectrometer operating at the X-band frequency (9.4 GHz) to investigate the oxygen vacancies at room temperature. The proportion of different valence elements in the sample was obtained by X-ray photoelectron spectroscopy (XPS, Thermo Scientific, ESCALab250, United States) test. The ^57^Fe Mössbauer spectra were recorded on a SEE Co W_3_O_4_ Mössbauer spectrometer, using a^57^Co/Rh source in transmission geometry. The data were fitted by using the MössWinn 4.0 software. The content of the different valent Fe was determined from the spectra.

### 2.3 Removal of Pb^2+^

Batch adsorption experiments were carried out to remove Pb (II) using magnetite and its calcined products by changing the initial Pb (II) concentration and contact time at a fixed amount of adsorbent dosage of 5 g/L. A mass of 0.1 g magnetite and a volume of 20 mL Pb (II) solutions were used in all batch experiments. They were mixed in 50 mL centrifuge tubes in duplicates for each condition. The stock solution was diluted to obtain the standard solution of concentrations 5, 10, 20, 40, 80, 120, 160, and 200 mg/L. The pH of the solution was adjusted to 5.5, which was consistent with the pH of the natural environment without precipitation. The mixture of adsorbent and solution was shaken on a reciprocal shaker at 150 rpm for 24 h. After being centrifuged for 10 min at 8,000 rpm, the suspension was analyzed for equilibrium Pb (II) concentration. With an initial Pb (II) concentration of 200 mg/L, the mixture was shaken on a reciprocal shaker at 150 rpm for 0.1, 0.5, 1, 2, 3, 6, 9, 12, and 18 h. The suspensions were then analyzed for equilibrium Pb (II) concentrations at these specific times. The residual Pb (II) concentration in the solution was determined with ICP.

## 3 Results and discussion

### 3.1 Characterization of magnetite and calcined products

**FIGURE 1:**
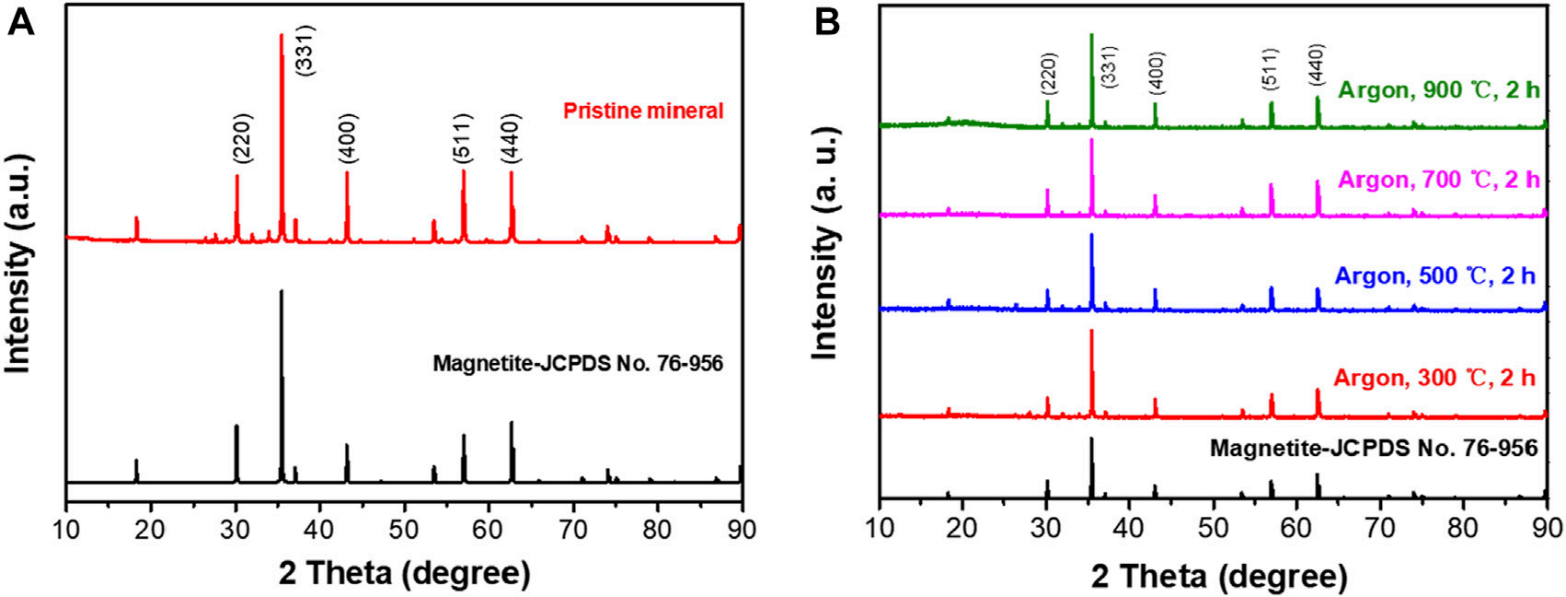
XRD patterns of magnetite ore **(A)** and its calcined products in argon atmosphere **(B)**.

The magnetite and its calcined products were analyzed by XRD, and the results are shown in Figure 1. The natural magnetite is mainly composed of Fe_3_O_4_ (Figure 1A), which is consistent with the diffraction pattern of Magnetite (JCPDS: 76-956). The content of each element in mineral materials was tested by XRF. The results were listed in Table 1, which were almost 97% iron oxide. It showed that the magnetite was of high purity, and some weak peaks not assigned in the XRD spectra might be caused by some very trace impurities in the raw ore, which were ignored in the experiment.

**TABLE 1:** X-Ray Fluorescence results of pristine magnetite.

Element	Fe	O	Mg	Si	Al	C	Ca	Other
Result (mass%)	69.9	27.5	0.966	0.427	0.270	0.588	0.117	0.142

After calcination in argon, the XRD patterns are shown in Figure 1B. The product is still magnetite after calcined at 300°C, 500°C, 700°C and 900°C in Ar for 2 h, which has apparent characteristic diffraction peaks at 30.16°, 35.51°, 43.12°, 57.01° and 62.59°. All the diffraction peaks can be indexed to the standard pattern of magnetite, and no new substances were generated.

**FIGURE 2:**
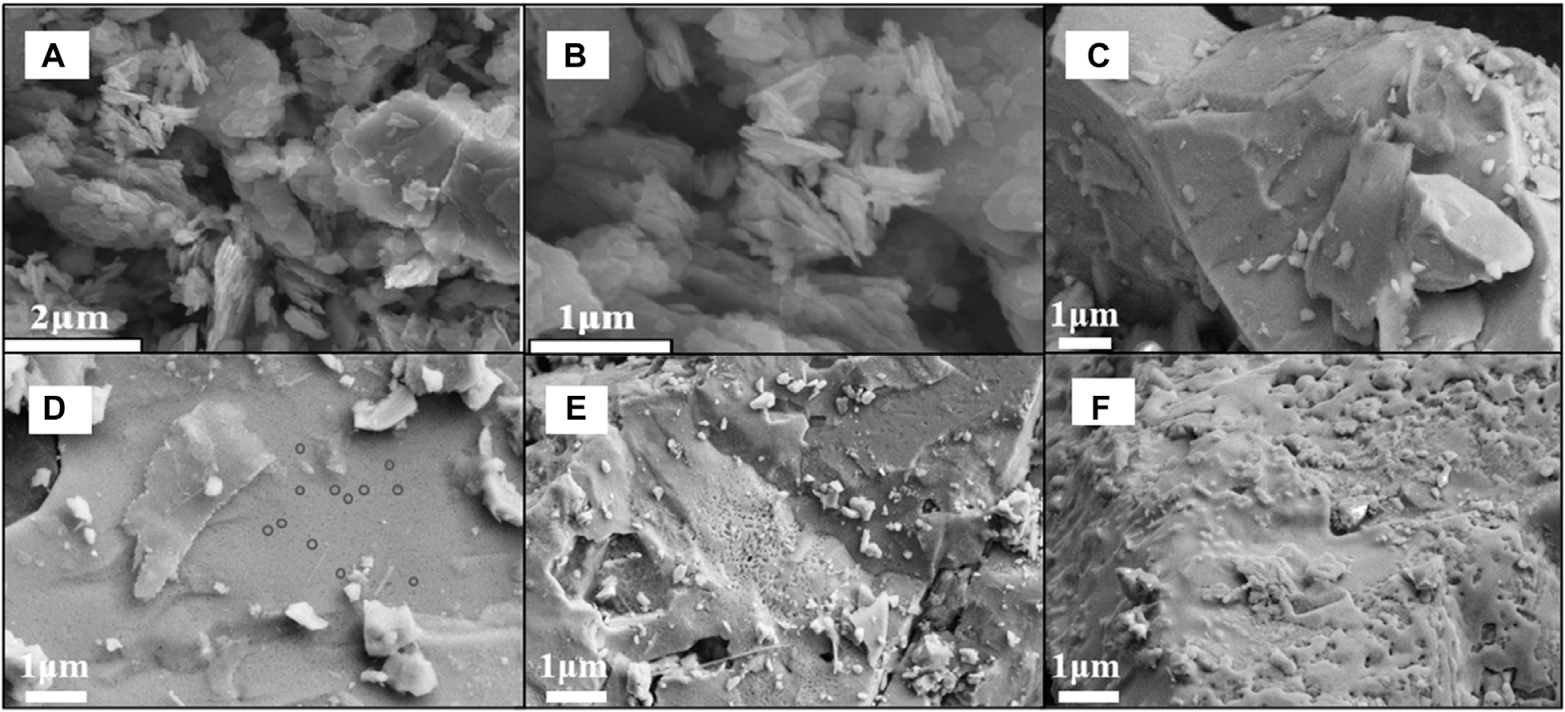
SEM images of magnetite **(A, B)** and its calcined products in argon at 300°C **(C)**, 500°C **(D)**, 700°C **(E)**, 900°C **(F)**.

SEM analysis was carried out so as to explore the effect of heat treatment at different temperatures on the morphology of magnetite, and the pictures of natural magnetite and its calcined products in argon are shown in Figure 2. The information we can get from the figure is that the size of the untreated magnetite is mostly 1∼5 μm, without any pores on the surface. As the calcination temperature increased in argon, the surface of the sample gradually changed, as shown in Figures 2C–F, which indicated the morphology of the samples at different temperatures.

As the temperature rised, some nano-pores are gradually formed on the surface of the mineral. At 300°C, the surface was still smooth without morphological changes; at 500°C, the surface had small deformation, and there were some tiny pits; at 700°C, the surface micropores gradually became larger; at 900°C, the surface melted and blocked the pores.

**FIGURE 3:**
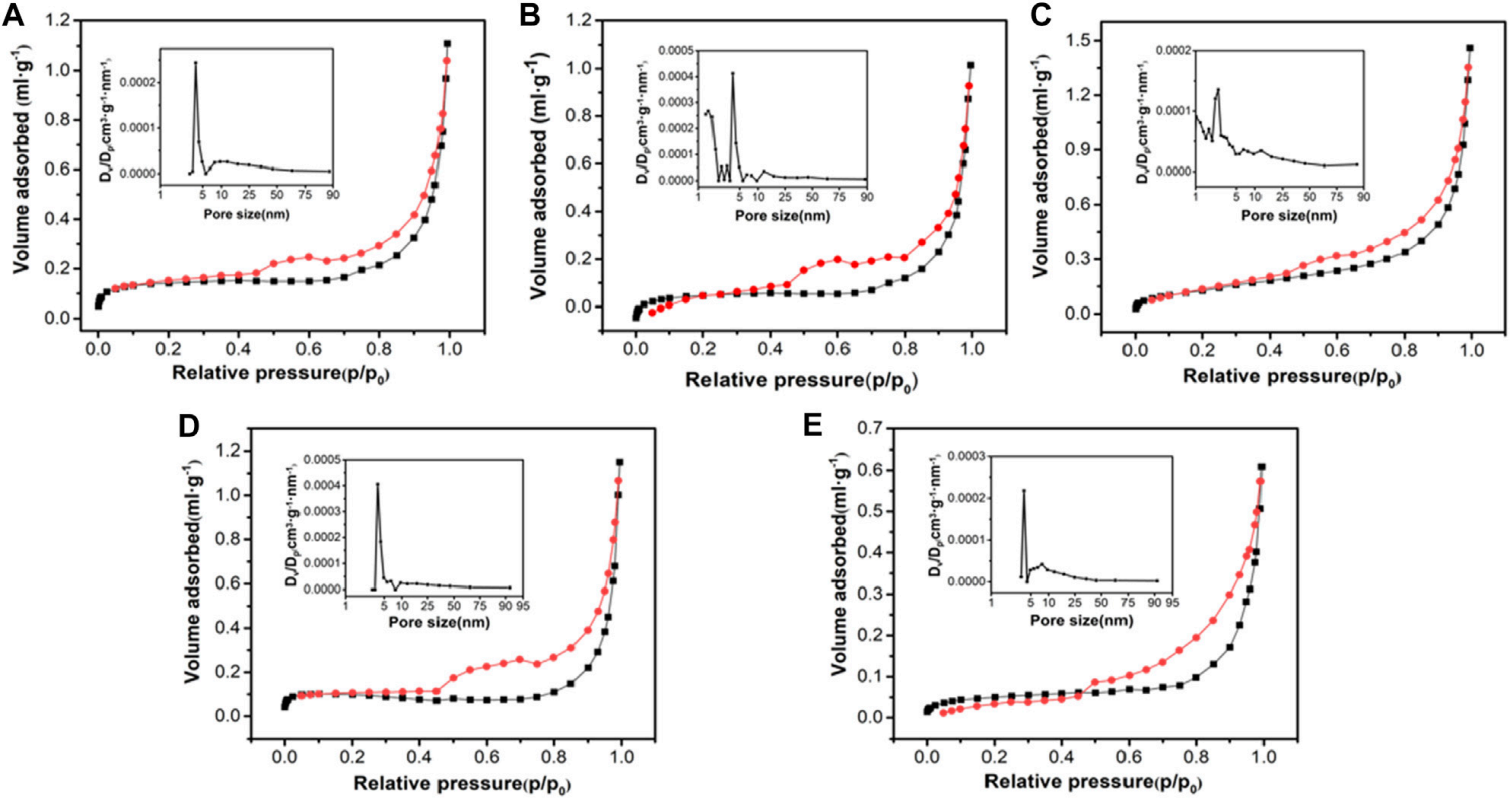
N_2_ adsorption-desorption isotherms and pore size distribution curves of Mag-25 **(A)**, Mag-300 **(B)**, Mag-500 **(C)**, Mag-700 **(D)**, and Mag-900 **(E)**.

In general, the surface area of the adsorbent affected its adsorption performance, so the specific surface area of this series of materials was tested. As shown in Figure 3, the specific surface areas of Mag-25, Mag-300, Mag-500, Mag-700, and Mag-900 were 0.53, 0.52, 0.42, 0.57, and 0.28 m^2^ g^-1^, respectively. When the temperature is below 700°C, the specific surface area of the sample had no noticeable change. Once the temperature reaches 900°C, the specific surface area dropped sharply to 0.28, which was also consistent with the conclusion of SEM. High temperature resulted in the surface melting of magnetite, thus leading to the decrease of specific surface area.

### 3.2 Adsorption isotherm

**FIGURE 4:**
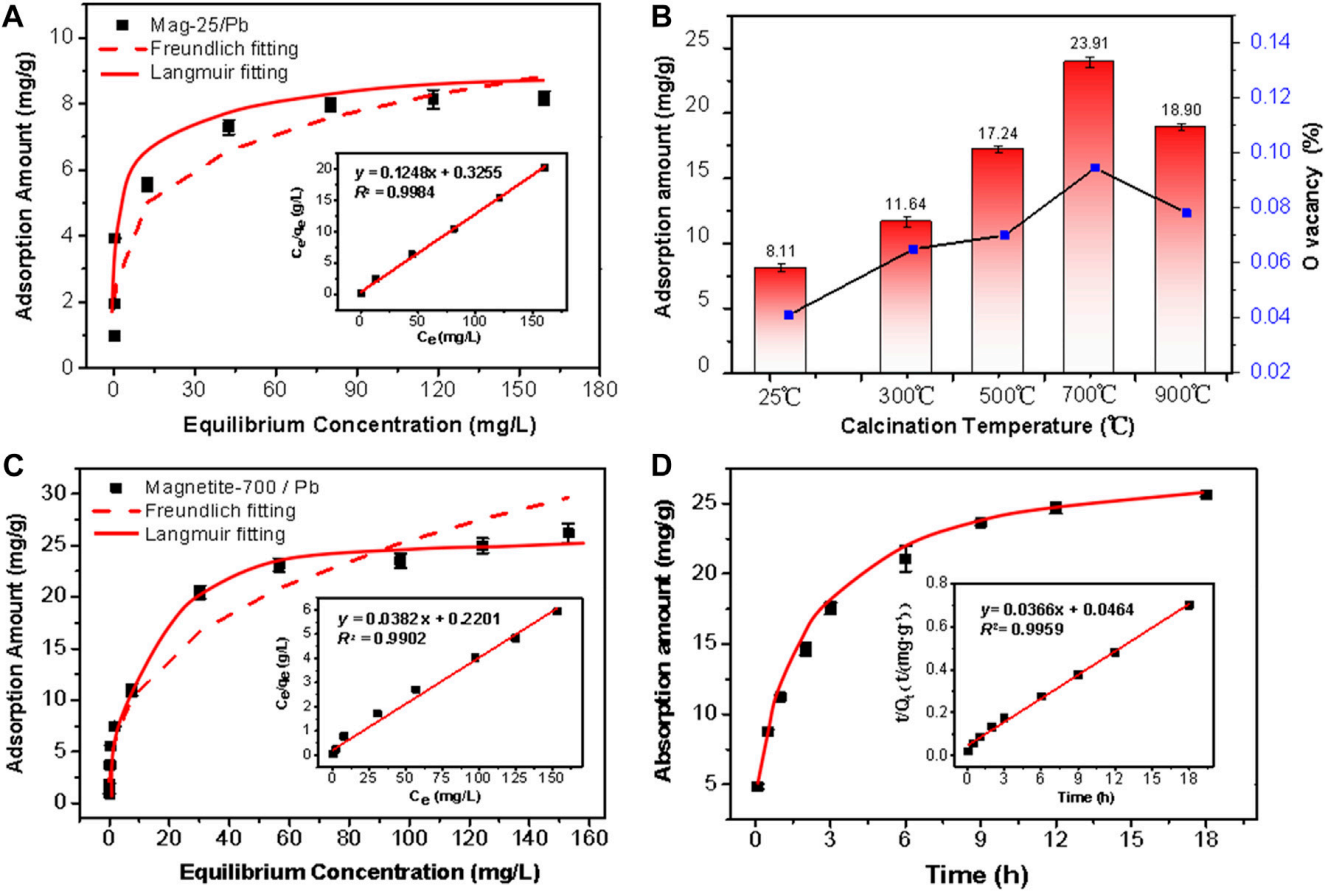
Pb(II) adsorption by **(A)** Mag-25, **(B)** magnetite at different calcination temperatures and the correlation with Oxygen vacancy, **(C)** Mag-700. The data were fitted to the Langmuir (solid line) and Freundlich (dashed line) isotherm models **(A, C)**. The insert is Langmuir adsorption isotherms model fitting. **(D)** Kinetics of Pb(II) adsorption by Mag-700 fitted to the pseudo-second-order kinetics (solid line). The insert is the linear plot of the pseudo-second-order reaction.

The raw magnetite had poor adsorption performance on Pb (II). The adsorption capacity was about 8 mg/g and accorded with the Langmuir adsorption model (Figure 4A). To improve its adsorption performance, the raw minerals were treated at different temperatures to obtain a series of samples. The effect of pH on Pb (II) adsorption was vital. The adsorption of Pb (II) onto magnetite was found to be enhanced as pH increased (Trivedi et al., 2003; Liang et al., 2017). This was because the deprotonation of magnetite is promoted in alkaline solutions. Pb (II) adsorption onto magnetite was favored when the surface charge of the mineral was negative (Bradl, 2004; Mamindy et al., 2009). The initial concentration of Pb (II) was 200 mg/L, and the experiment was conducted at pH 5.5 to eliminate the impact of precipitation on the removal rate at a high pH (Karami, 2013). In Figure 4B, the adsorption capacity of Mag-25 was the lowest among the series of materials. As the increase in calcination temperature, the adsorption performance of the material was significantly improved and reached its highest when the calcination temperature reached 700°C. Subsequently, the adsorption performance of Mag-900 decreased slightly. (The correlation between adsorption capacity and oxygen vacancy content would be discussed in Section 3.4.3) Mag-700 was selected for the next adsorption experiment of Pb (II). The adsorption capacity and affinity of the adsorbent were obtained by adsorption isotherm. The amount of Pb (II) ion adsorbed per unit of adsorbent at equilibrium could be gained by isotherm study. Langmuir and Freundlich isotherm models were used to analyze the batch experimental data with initial Pb (II) concentrations ranging from 5 to 200 mg/L at pH ≈ 5.5.

The Langmuir isotherm applies to the monolayer adsorption sites on an energetically homogenous surface with a finite number of identical sites (Nemr et al., 2008). No further adsorption can occur once a site is occupied (Yuan et al., 2013). The Freundlich isotherm is an empirical equation describing a heterogeneous system (Qiu et al., 2015; Abebe et al., 2021). The linear forms of Langmuir and Freundlich isotherm models are described by Equations 1, 2:

$$\frac{C_{e}}{q_{e}}=\frac{1}{q_{\max}b}+\left(\frac{1}{q_{\max}}\right)C_{e}$$ (1)

$$\ln q_{e}=\ln K_{f}+\frac{1}{n}\ln C_{e}$$ (2)

where C_e_ and q_e_ are the equilibrium concentration (mg/L), and the amount of Pb (II) adsorbed per Gram of adsorbent (mg/g), respectively. The q_max_ and b are the adsorption capacity of the adsorbent (mg/g) and the Langmuir affinity constant (L/mg) of the binding sites, respectively. K_f_ (mg/g) and n (g/L) are the empirical Freundlich constants representing the bond strength and the adsorption intensity of the heterogeneous surface. As can be seen from Table 2, the adsorption reaction was favorable as the n was 2.61, which lied between 1 and 10 (Zhao et al., 2013). Besides the above findings, the experimental *R* ^2^ value (0.9902) of the Langmuir model was better (Figure 4C). The essential features of Langmuir isotherm can be expressed in terms of dimensionless constant known as separation factor or equilibrium parameter (R_L_) using Eq. 3. The value of R_L_ between 0 and 1 indicates favorable adsorption; R_L_ > 1 indicates unfavorable adsorption; R_L_ = 1 represents the linear adsorption, and the adsorption operation is irreversible if R_L_ = 0 (Arulkumar et al., 2012).

$$R_{L}=\frac{1}{1+bC_{0}}$$ (3)

where b (L/mg) and C_0_ (mg/L) are the Langmuir affinity constant and initial concentration of Pb (II). Table 2 indicated the positive value of R_L_ is 0.03 and it is within 0 and 1. Thus, it indicated the adsorption was favorable and also feasible onto Mag-700.

**TABLE 2:** Comparison of isotherm models of Pb (II) adsorption by Mag-700 at different concentrations.

*T*/K	Langmuir			Freundlich		
	*q*_max_/mg g^-1^	*b*	*R* ^*2*^	ln*K*_*f*_	*n*	*R* ^*2*^
293	26.18	0.1735	0.9902	0.9215	2.61	0.9283

Thus, a conclusion could be drawn that the adsorption of Pb (II) onto Mag-700 was not a multi-molecular adsorption process, and its equilibrium adsorption capacity was 26.21 mg/g. Compared with other magnetite materials, the adsorption performance of lead is even better than that of nanoparticle magnetite (Table 3) (Wang et al., 2010; Wang et al., 2014; Kumari et al., 2015; Liang et al., 2017; Lankathilaka et al., 2021). According to the adsorption and BET test results of the series of materials, the improvement of adsorption performance does not correlate with the specific surface area.

**TABLE 3:** Comparison of Pb (II) adsorption properties of magnetite in different references.

Adsorbent	Q_0_ (mg/g)	Surface area (m^2^/g)	pH	Concentration (mg/L)	Dosage (g/L)	References
Magnetite nanoparticles	22.9	115.3	5	0.5–5 (mmol/L)	10	Wang et al. (2010)
Magnetite nanoparticles	20.14	125.77	3.8	10–100	4	Wang et al. (2014)
Magnetite nanospheres	19	11.3	5	0–100	2	Kumari et al. (2015)
Magnetite	9.8	28	5	10–160	2	Liang et al. (2017)
Magnetite/kaolin granules	0.9	—	—	10–100	5	Lankathilaka et al. (2021)
Calcined magnetite	26.21	0.57	5.5	5–200	5	This work

### 3.3 Adsorption kinetics

In the research field of solid-liquid adsorption, the pseudo-first-order (Lagergren, 1998) and pseudo-second-order kinetic models (Ho and McKay, 1999) are applied to the adsorption data obtained. The integral forms of these two models are expressed using the following Eqs 4, 5:

$$\ln\left(q_{e}-q_{t}\right)=\ln q_{e}-k_{1}t$$ (4)

$$\frac{t}{q_{t}}=\frac{1}{k_{2}{q_{e}}^{2}}+\frac{1}{q_{e}}t$$ (5)

where *q*_*e*_ (mg/kg) is the amount of Pb (II) adsorbed at equilibrium, and *q*_*t*_ (mg/kg) is the amount of Pb (II) adsorbed on adsorbent at any time. *k*_*1*_ (1/min) is the rate constant of the adsorption reaction. *k*_*2*_ (g/mg/min) is the rate constant of the second-order reaction.

The kinetic model parameter values and regression coefficients (*R* ^*2*^) obtained from each kinetic plots (Figure 4D) are given in Table 4. The value of *R* ^*2*^ of the pseudo-second-order kinetic model was relatively higher (0.9959) than that of the pseudo-first-order model (0.9327). Therefore, the adsorption of Pb (II) onto Mag-700 could be described in terms of the pseudo-second-order kinetics. The adsorption equilibrium was reached after 9 h.

**TABLE 4:** Comparison of kinetic parameters of Pb (II) adsorption onto Mag-700.

*C*_*0*_ (mg/L)	*q*_*e(exp)*_ (mg/g)	Pseudo-first-order kinetic model			Pseudo-second-order kinetic model		
		*q*_*e(cal)*_ (mg/g)	*K*_*1*_ (1/h)	*R* ^*2*^	*q*_*e(cal)*_ (mg/g)	*K*_*2*_ (g/mg/h)	*R* ^*2*^
200	26.21	27.20	0.378	0.9327	27.32	0.029	0.9959

### 3.4 Mechanism

Ions diffuse in metal oxides through point defects. According to the vacancy mechanism, vacancies jump into neighboring lattice sites, and ions on the sites jump in reverse (Nakamura et al., 1978). The magnetite in this work is very likely to form such defects after high-temperature calcination, which may also be the reason for the improvement of adsorption performance. Therefore, XRF, EPR, Rietveld refinement, Mössbauer spectrum, and XPS were used to verify the adsorption mechanism of magnetite.

First, according to the XRF results in Table 1, more than 97% was iron oxide. In addition, there were trace impurities such as magnesium, silicon, and aluminum. However, in the crystal structure of magnetite, the Mg may replace some of the Fe^2+^, given the relatively high magnesium content of the impurity. It thus affected its charge distribution, so the influence of magnesium was taken into account when calculating the chemical formula. Based on the atomic ratio of Fe, O, and Mg, the chemical formula of magnetite was determined as Fe_2.90_Mg_0.09_O_4_ if there was no oxygen vacancy.

#### 3.4.1 Crystal structure

**FIGURE 5:**
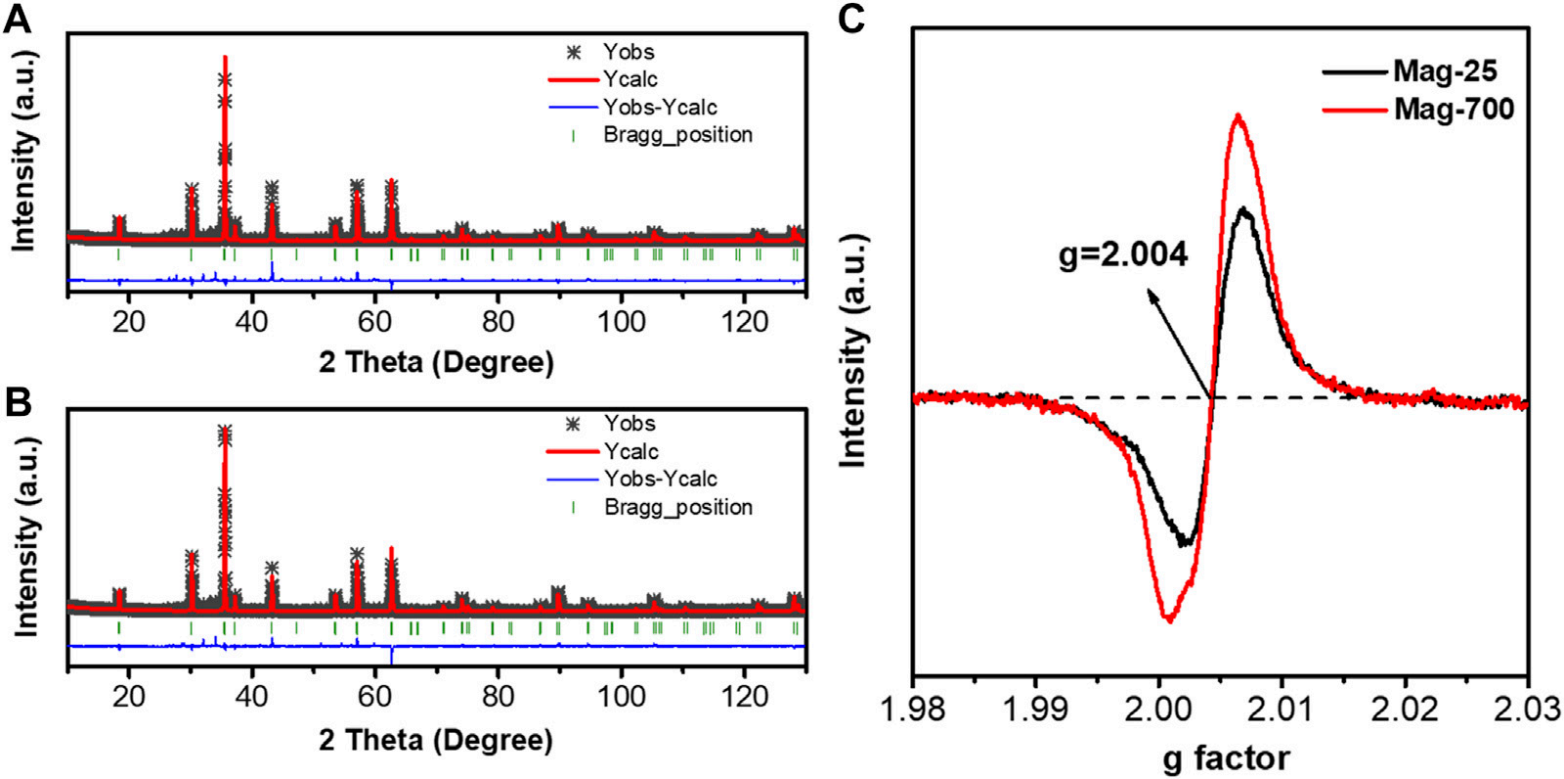
Rietveld refinement of the XRD patterns for **(A)** Mag-25; **(B)** Mag-700 and EPR spectra measured at room temperature **(C)**.

To further identify the occupancies of iron and oxygen elements in the crystal structure of magnetite, these two samples Mag-25 and Mag-700, were used for the XRD Rietveld refinement, performed using the TOPAS V7.11 package (Bruker, 2009). The structure model was acquired using the JCPDS No.76-956 card. Figures 5A, B show the Rietveld refinement of Mag-25 and Mag-700, where the black asterisk, solid red line, short green vertical line, and solid blue line in the bottom represent the XRD measurement data, the calculated pattern, the Bragg position, and the distinction between the observed and the calculated patterns, respectively. The refinement parameters for magnetite obtained from the Rietveld refinement are shown in Table 5. The final refinement was stable and convergent well with low residual factors *R*_*p*_ = 10.07%, *χ* ^*2*^ = 3.05 and *R*_*p*_ = 8.78%, *χ* ^*2*^ = 2.60, indicating no unidentified diffraction peaks due to impurity. The occupancies of Fe (oct) were almost constant, while the Fe (tet)’s declined obviously (Table 6). And the occupancies of O also declined from 0.987 to 0.978. The occupation fraction from Rietveld refinement illustrates that Fe cation vacancies in tetrahedrons and O vacancies are generated in Mag-700.

**TABLE 5:** Magnetite refinement parameters were obtained from the Rietveld refinement using X-ray powder diffraction data at room temperature.

Samples	Mag-25	Mag-700
Space group	Fd3m	Fd3m
Crystal system	Cubic	Cubic
*a/b/c* (nm)	8.397865 (55)	8.398043 (46)
*α/β/γ*	90°	90°
*V* (Å^3^)	592.252 (12)	592.290 (10)
*R-Bragg*	8.18920665	6.85563089
*R*_exp_	14.66	13.24
*R*_*wp*_	4.81	5.10
*R*_*p*_	10.07	8.78
*χ* ^*2*^	3.05	2.60

**TABLE 6:** The occupancy parameters of Mag-25 and Mag-700.

Samples	Occ-Fe (tet)	Occ-Fe (oct)	Occ-O	beq
Mag-25	1	0.995	0.987	14.66
Mag-700	0.986	1	0.978	13.24

The single-featured EPR spectra have been identified as oxygen vacancy at g = 2.004 (Fan J, et al., 2020), so the EPR was applied to determine further the number of O vacancies in Mag-25 and Mag-700, which was shown in Figure 5C. The value of the free electron *g*-factor varies with the nature of the defect and the surroundings, and it is usually calculated using quantum electrodynamic methods (Lu D, et al., 2022). Only one type of signal (*g* = 2.004) was exhibited in Mag-25 and Mag-700 samples arising from the O vacancies. For Mag-25, the weak peak reflected a small number of vacancies. The signal for Mag-700 significantly intensified and showed 1.6 times that of mag-25, indicating elevated content of O vacancies in Mag-700.

#### 3.4.2 Mössbauer spectroscopy

**FIGURE 6:**
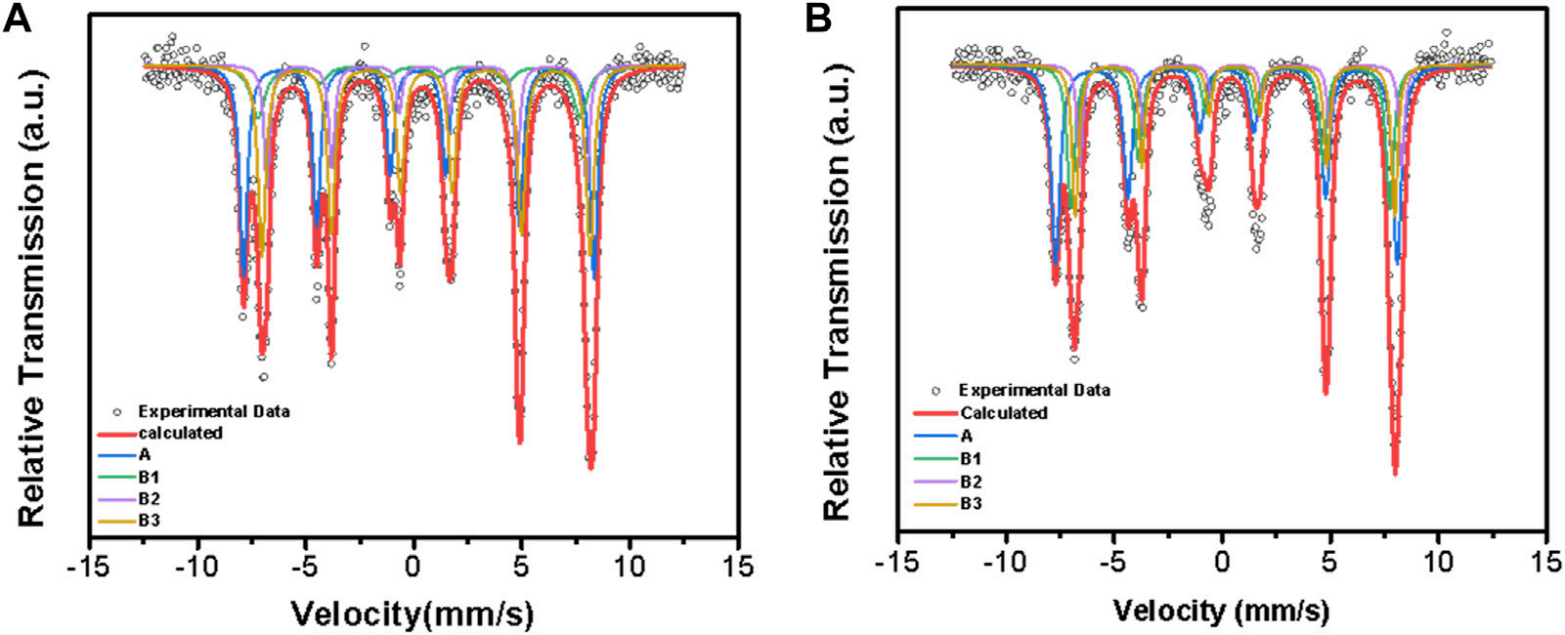
Room temperature Mössbauer spectra of Mag-25 **(A)** and Mag-700 **(B)**. The black circles stand for the experimental data, and the thick red line stands for the calculated data, Fe^3+^ **(A)** sextet is shown in blue, Fe^3+^ (B1), Fe^2+^ (B2) and Fe^2+^ (B3) sextets are shown in blue, green, purple and yellow, respectively.

Also, the change of the occupation of Fe^2+^ and Fe^3+^ in magnetite needs to be confirmed to determine the structural formula of magnetite, so the Mössbauer spectrum tests were carried out for Mag-25 and Mag-700. The fitted spectra for the lowest and highest temperatures are shown in Figure 6, and the hyperfine parameters in the form are shown in Table 7.

**TABLE 7:** Parameters of the hyperfine structure.

Sample	Position	IS (mm/s)	QS (mm/s)	H (T)	*Γ* (mm/s)	Area (%)	Valence-state
Mag-25	A	0.21	0.03	50.40	0.42	38.5	8×Fe^3+^
	B1	0.11	0.26	46.36	0.81	28.7	8×Fe^3+^
	B2	0.54	0.11	45.96	0.33	11.1	5×Fe^2+^
	B3	0.57	−0.02	47.22	0.43	21.7	3×Fe^2+^
Mag-700	A	0.19	−0.03	49.12	0.48	36.9	8×Fe^3+^
	B1	0.38	−0.01	45.86	0.43	26.8	8×Fe^3+^
	B2	0.76	0.18	46.27	0.31	13.1	5×Fe^2+^
	B3	0.57	0.03	45.92	0.35	23.2	3×Fe^2+^

Note: IS, means isomer shift; QS, means quadrupole splitting, H stands for the hyperfine magnetic field, and *Γ* stands for line broadening.

The electron charge density of the iron nuclei is represented in the Mössbauer spectrum as an isomer shift relative to the metal bcc α-Fe as a reference substance. The nuclear charge densities of 8 non-equivalent A sites and 16 non-equivalent B sites can be determined by the calculation of DFT (Řezníček et al., 2015; Řezníček et al., 2017; Chlan et al., 2018). The four sextets correspond to the tetrahedral (A) sites and the octahedral (B) magnetic sublattice, and the spectral analysis was based on those considerations (Sorescu et al., 1998; Sorescu et al., 2003). The trimeron model adequately described the electronic structure and grouped the B sites in an 8:5:3 ratio (Senn et al., 2011; Řezníček et al., 2015). The first group contains Fe^3+^-like ions, defined as B1, while the other groups are occupied by Fe^2+^-like ions. C (Řezníček et al., 2017).

The area ratios of the four groups of lines A, B1, B2, and B3 represent the ratios of ions at different positions. There is a decrease in the proportion of the Fe^3+^ at positions A and B1 of the two materials before and after the calcination of magnetite while the area ratio of B2 and B3 increased, which shows that the content of Fe^2+^ in the sample is relatively increased after calcination at 700 °C. Since the oxidation and reduction of magnetite are carried out in the range of its non-stoichiometric composition, vacancies will also appear in the materials (Nakamura et al., 1978). The non-stoichiometric characteristics of magnetite are the presence of oxygen vacancy in the structure. This is due to the rearrangement of the internal Fe atoms. The proportion of Fe^2+^ in the octahedron increases, and the content of Fe^3+^ in the tetrahedron and octahedron decreases, resulting in an imbalance of charge that removes additional O atoms from the structure. From the ratio of Fe^2+^ to Fe^3+^ in the Mössbauer spectrum and the results of Rietveld refinement, the structural formulae of Mag-25 and Mag-700 can be written as Fe^3+^[Fe^2+^_0.951_Mg_0.090_Fe^3+^_0.949_]O_3.965_ and Fe^3+^_0.990_ [Fe^2+^_1.053_Mg_0.090_Fe^3+^_0.857_]O_3.914_, respectively. It can be seen that there are oxygen vacancies in magnetite, which was consistent with Rietveld refinement results.

#### 3.4.3 XPS analysis

XPS is considered a highly efficient and reliable method for determining the ratio of Fe^2+^ to Fe^3+^ on active surface atoms (Liu et al., 2016). In order to explore the reason why the adsorption effect of Mag-700 was stronger than that of raw magnetite, XPS analysis was carried out on raw magnetite and a series of materials calcined at different temperatures to study the effect of calcination in argon on the valence state of iron in the mineral.

**FIGURE 7:**
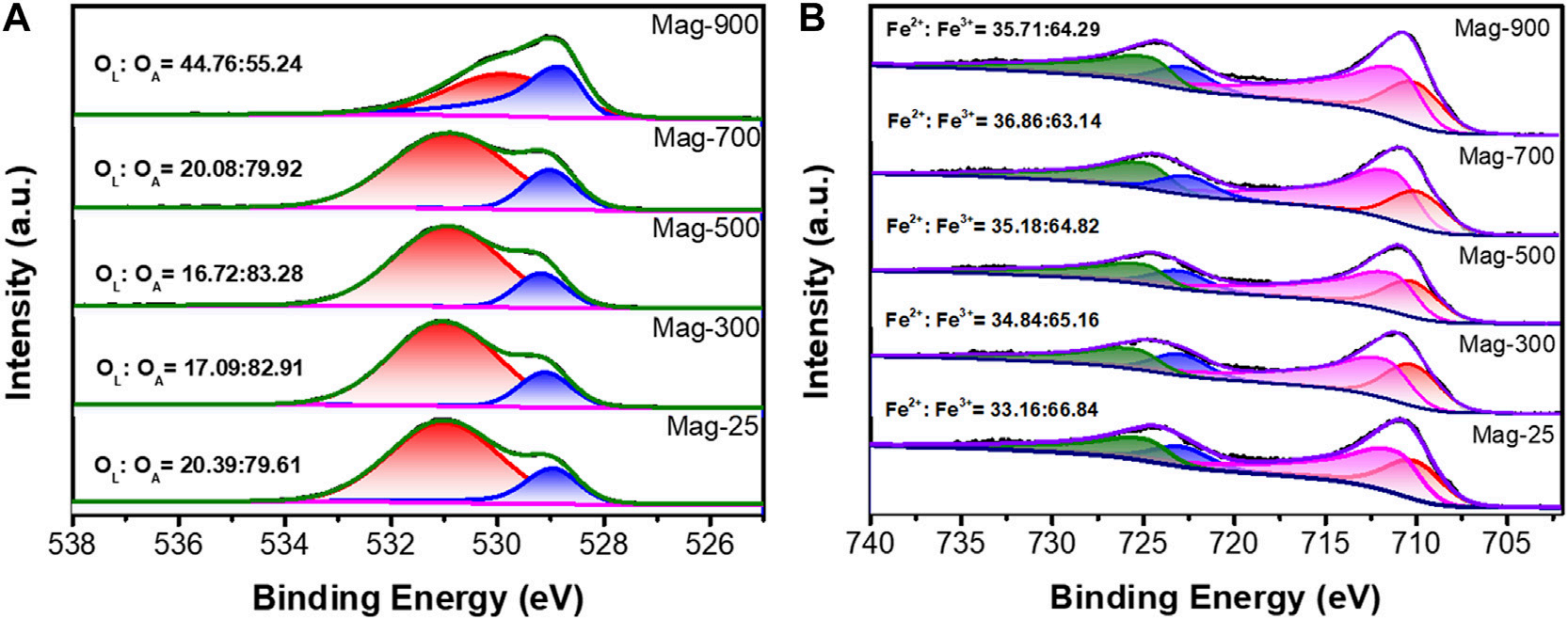
O 1s XPS spectra **(A)** and Fe 2p spectra **(B)** of magnetite at different temperatures.

Surface composition and valence states of O and Fe have been investigated by X-ray photoelectron spectroscopy, as shown in Figures 7A, B. The binding energy at 531.8 eV was attributed to surface adsorbed oxygen (O_A_), and the photoelectron peak of the surface lattice oxygen (O_L_) had binding energy at around 529 eV (Deng et al., 2016). The intensity of the O_L_ peak decreased to the minimum when the calcination temperature was up to 500 °C and then increased with the temperature further rising. In Mag-900, the ratio of O_L_/O_A_ increased to 44.76/55.24. The increase in O_L_ peak intensity may be due to the oxygen vacancies caused by the high temperature (Chen et al., 2017). When the calcination temperature was above 700 °C, the relative content of lattice oxygen in the sample increased sharply, which may be caused by the release of the surface adsorbed oxygen at higher temperatures.

As shown in Figure 7B, Fe 2p3/2 of Fe_3_O_4_ has no satellite peak, and distinct two peaks of Fe2p with binding energies at 710.7 and 724.2 eV were assigned to spin-orbit peaks Fe 2p3/2 and Fe 2p1/2 of Fe_3_O_4_ respectively (Yamashita and Hayes, 2008; Farghali et al., 2015). The main peaks of Fe^3+^ 2p1/2 and Fe^3+^ 2p1/2 are located at 711.7 eV and 725.3 eV (Wang et al., 2017). Moreover, by fitting Fe 2p double peak, another two peaks yield at 710 eV and 724 eV, which belongs to Fe^2+^ 2p3/2 and Fe^2+^ 2p1/2 (Hou et al., 2016; Jia et al., 2018; Li et al., 2020a). The peak position does not shift significantly with the temperature change. When the temperature increases to 700°C, the proportion of Fe^2+^ increases from 33.16% to 36.86%, and the content of Fe^3+^ decreases by reducing to Fe^2+^. However, its ratio decreases when the temperature rises to 900°C, and the oxidation reaction plays a dominant role.

**FIGURE 8:**
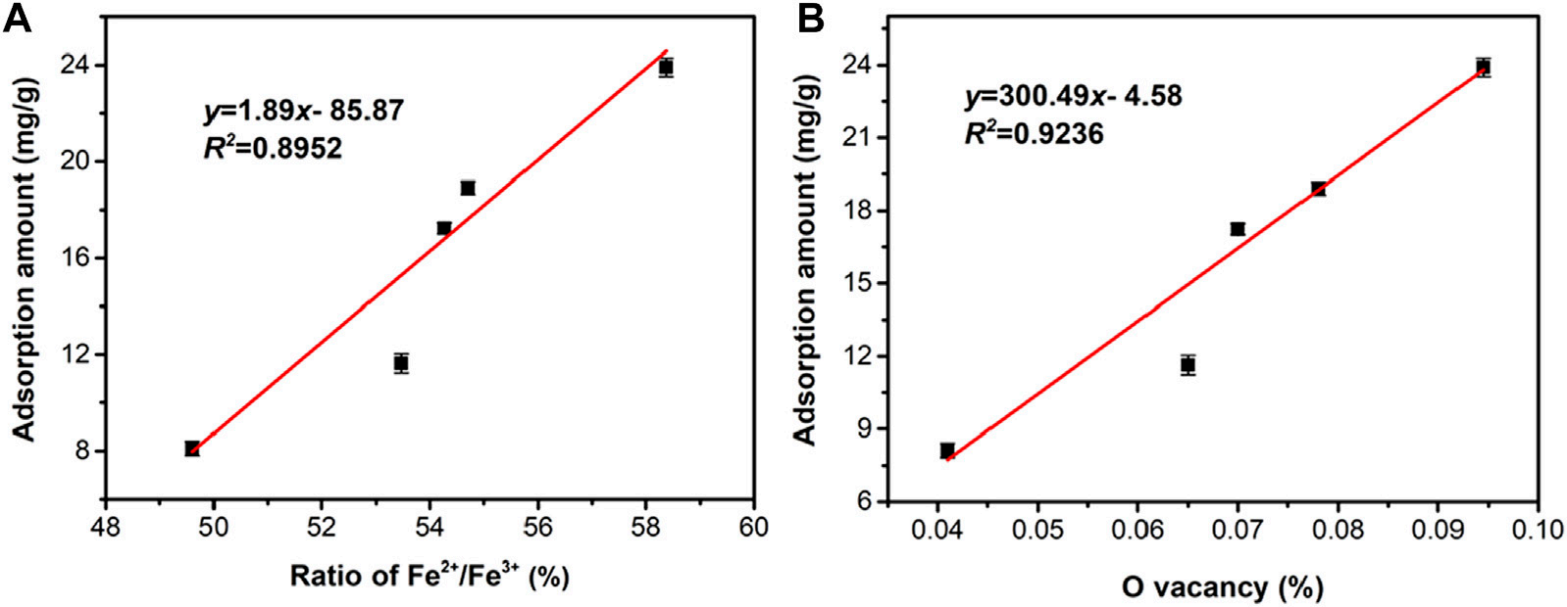
Correlation analysis of adsorption capacity with **(A)** proportion of Fe^2+^; and **(B)** oxygen vacancy content in series magnetite.

In light of these results, it can be concluded that heating magnetite under an inert atmosphere will cause the appearance of oxygen vacancies, which leads to the increase of the proportion of Fe^2+^ to maintain the overall charge balance. However, the ratio of Fe^2+^ in the Mag-900 sample is abnormal, which may be due to the oxidation of the sample at a very high temperature. The total amount of Fe did not change according to Fe occupancy in Rietveld refinement. The chemical formula for the surface of magnetite can be obtained from the ratio of Fe^2+^/Fe^3+^ in XPS. Table 8 shows that there are Fe and O vacancies in the pristine Mag-25, and the amount of O vacancies increased to a maximum in Mag-700, then decreased slightly in Mag-900. In the chemical formulae obtained from XPS results, the variation trend of oxygen vacancy content is consistent with the experimental results of Pb (Ⅱ) removal (Figure 4B), indicating that oxygen vacancy plays a significant role in the adsorption reaction. In addition, the adsorption amount of Pb (Ⅱ) has a good correlation with the proportion of Fe^2+^ (Figure 8A) and the content of oxygen vacancy content (Figure 8B) in magnetite. The introduction of oxygen vacancy can be the result of the rearrangement of iron atoms, which directly improves the adsorption capacity of magnetite to Pb (Ⅱ).

**TABLE 8:** Related content of Fe^2+^ and Fe^3+^ in series materials and the given structural formulae.

Samples	Fe 2p 1/2 (eV)		Fe 2p 3/2 (eV)		Atom ratio Fe^2+^/Fe^3+^	Chemical formulae
	710	723	711.7	725.3		
Mag-25	24.25	8.91	43.49	23.35	33.16/66.84	Fe^2+^_0.962_Fe^3+^_1.983_Mg^2+^_0.090_O_3.959_
Mag-300	24.63	10.21	47.63	17.54	34.84/65.16	Fe^2+^_1.010_Fe^3+^_1.890_Mg^2+^_0.090_O_3.935_
Mag-500	24.87	40.34	12.87	21.90	35.18/64.82	Fe^2+^_1.020_Fe^3+^_1.880_Mg^2+^_0.090_O_3.930_
Mag-700	24.52	12.37	46.89	16.30	36.86/63.14	Fe^2+^_1.069_Fe^3+^_1.831_Mg^2+^_0.090_O_3.9055_
Mag-900	24.53	11.18	44.64	19.65	35.17/64.29	Fe^2+^_1.036_Fe^3+^_1.864_Mg^2+^_0.090_O_3.922_

#### 3.4.4 Surface complexes

The mechanism of magnetite adsorption for Pb (Ⅱ) has been widely studied. It has been suggested that Pb (Ⅱ) is adsorbed onto the surface of magnetite by electrostatic attraction and forms surface complexes with the functional group (≡FeOH) generated by the protonation and deportation reactions (Giraldo et al., 2013; Wang et al., 2014). However, according to the pH_pzc_ of the magnetite, magnetite is positively charged, and the adsorption of Pb (II) should not depend on electrostatic bonding, mainly through the complexation of Pb (Ⅱ) with deprotonated surface hydroxyl groups. Pb (Ⅱ) acts as Lewis acid while the functional surface hydroxyl group (≡FeOH) serves as the Lewis base in deprotonated form (≡FeO-) to bind the Lewis acid Pb (Ⅱ) cation (Liang et al., 2014). Pb (Ⅱ) ions were mainly adsorbed on the surface of magnetite by inner-sphere complexation, and the adsorbed Pb (Ⅱ) species showed a bidentate binuclear corner-sharing geometry (Liang et al., 2017). The inner-sphere complexes are more stable than the outer-sphere complexes. This is because the former has the coordination covalent bonds as the main force, while the latter has an electrostatic bond as the dominant force. As Lewis basis, the hydroxyl group on the surface of magnetite is a critical functional group for adsorbing Pb (Ⅱ). In this work, the calcined Mag-700 has more defects, especially cationic vacancies and oxygen vacancies, which makes the magnetite exposed more functional groups and more reactive active sites (Li et al., 2014; Ali et al., 2021), and increases the contact field between the surface of magnetite and Pb (Ⅱ), which is the main reason for improving the adsorption performance of Pb (Ⅱ).

## 4 Conclusion

In conclusion, calcination in argon effectively increases the oxygen vacancy content in the crystal structure of magnetite, which plays a vital role in improving the removal of Pb (II). The results show that, compared with natural magnetite, high temperature significantly influences the defect density and affinity for Pb (II) of magnetite under low oxygen pressure. The defect contents and structural formulae of the magnetite samples were determined successfully through Rietveld refinement and the ratio of Fe^2+^ and Fe^3+^. In general, the defect degree of magnetite is linearly related to the removal capacity of Pb (II). Magnetite has a broad application prospect in wastewater remediation due to its simple defect engineering method, superior Pb (II) removal ability, rapid kinetics, and recyclability.

## Data Availability

The original contributions presented in the study are included in the article/supplementary material, further inquiries can be directed to the corresponding author.
